# Multiple brain infarctions and endomyocarditis in ANCA-negative eosinophilic granulomatosis with polyangiitis

**DOI:** 10.1016/j.heliyon.2023.e12881

**Published:** 2023-01-07

**Authors:** Takumi Tashiro, Urara Fujiwara, Yuichi Kira, Chihiro Karashima, Norihisa Maeda

**Affiliations:** a Department of Neurology, National Hospital Organization Beppu Medical Center, Japan; b Department of Cardiology, National Hospital Organization Beppu Medical Center, Japan

**Keywords:** Eosinophilic granulomatosis with polyangiitis, Vasculitis, Antineutrophil cytoplasmic antibodies, Multiple brain infarctions, Endomyocarditis

## Abstract

Eosinophilic granulomatosis with polyangiitis (EGPA) is a small vessel necrotizing vasculitis characterized by asthma and eosinophilia. Ischemic stroke is a rare complication of the disease. We herein report a case involving a 77-year-old woman with sinusitis who developed embolic stroke and splenic infarctions. Laboratory tests revealed hypereosinophilia and elevated troponin-T and N-terminal pro-brain natriuretic peptide. Antineutrophil cytoplasmic antibodies (ANCA) studies were negative. Skin biopsy showed infiltration of eosinophils into the arterial walls. These clinicopathological findings led to the diagnosis of EGPA. We also found the evidence of endomyocarditis as revealed by multimodality cardiac imaging. The patient underwent continuous immunosuppressive and anticoagulation therapy, and the infarctions did not recur. This report highlights the importance of histologically proven vasculitis with eosinophil infiltration and careful examination for cardiac involvement, especially in ANCA-negative patients.

## 1 Introduction

Eosinophilic granulomatosis with polyangiitis (EGPA) is a small vessel necrotizing vasculitis characterized by asthma and eosinophilia [1]. Peripheral neuropathy is frequent in EGPA patients, but central nervous system involvement is a rare complication [2]. Moreover, multiple organ infarctions, including brain infarctions, are extremely rare events. This report aims to present a case of ANCA-negative EGPA with multiple organ infarctions and to discuss the diagnostic approach for cardiac involvement.

## 2 Case report

A 77-year-old woman with a history of sinusitis was admitted to our hospital with acute right hemiparesis. On physical examination, the body temperature was 100 °F and purpura was present on her extremities. She had hemiparesis, sensory disturbance, and cerebellar ataxia of the right limbs. Blood tests showed a high eosinophil count of 10,864/μL (56.0% of white blood cell). Serum biochemistry also revealed an elevated erythrocyte sedimentation rate, IgE, troponin-T, and N-terminal pro-brain natriuretic peptide (NT-proBNP) concentration. All autoantibodies, including antineutrophil cytoplasmic antibodies (ANCA), were negative.

**Fig. 1:**
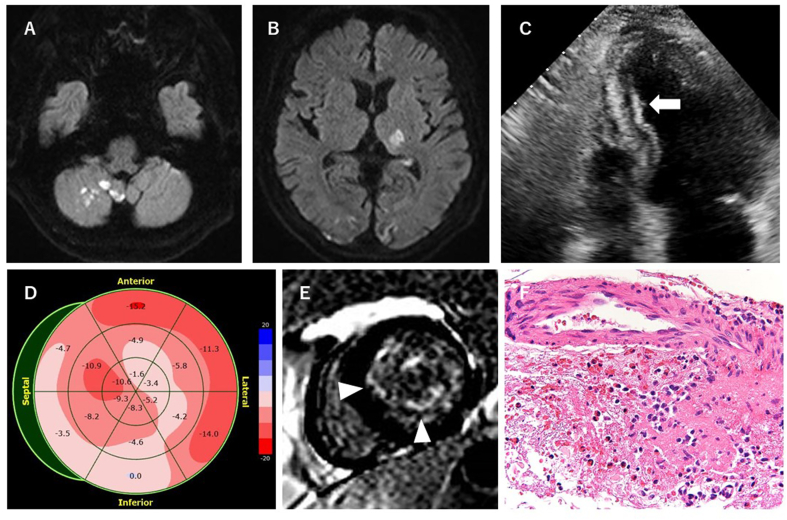
(A, B) Magnetic resonance imaging (MRI) upon admission. Diffusion-weighted MRI showed multiple infarctions in bilateral cerebrum and right cerebellum. (C) Transthoracic echocardiographic view. The high echogenicity of the endocardium in the interventricular septum and left ventricular inferior wall corresponded to areas of fibrosis (arrow). (D) The left ventricular global longitudinal strain was impaired in the interventricular septum and left ventricular inferior wall. (E) Cardiac MRI showed delayed enhancement in the interventricular septum and inferior endocardium (arrowheads). (F) Skin biopsy with hematoxylin and eosin staining revealed leukocytoclastic vasculitis accompanied by eosinophilic infiltration of the small vessels.

Brain magnetic resonance imaging (MRI) revealed multiple cerebral and cerebellar infarctions (Fig. 1A and B). MR angiography showed left posterior cerebral artery (PCA) occlusion. Computed tomography revealed splenic infarctions. Cerebrospinal fluid tests and a nerve conduction study were normal. Transthoracic echocardiography revealed increased endocardial brightness in the interventricular septum and left ventricular inferior wall, and the left ventricular global longitudinal strain (GLS) was impaired (Fig. 1C and D). Cardiac MRI showed delayed endocardial enhancement in the same area (Fig. 1E). Transesophageal echocardiography showed no obvious thrombus.

There was no evidence of hematologic malignancies and idiopathic hypereosinophilic syndrome. Skin biopsy showed eosinophilic vasculitis, consistent with EGPA (Fig. 1F). The patient received intravenous pulse methylprednisolone (1000 mg/day for 3 days) followed by oral corticosteroids (1 mg/kg/day), intravenous cyclophosphamide, and anticoagulation therapy. After intravenous pulse methylprednisolone, blood eosinophil count had rapidly decreased to 0/μL and serum markers of inflammation had returned to normal. Myocardial biopsy specimen obtained from the right ventricular wall demonstrated non-specific mild intercellular myocardial edema, and coronary angiography showed normal findings. A week later, both hemiparesis and sensory deficits ameliorated, then the patient was able to walk with one crutch. One month later, there were no recurrent infarctions with recanalization of the left PCA and remarkable improvement in mean GLS. The level of NT-proBNP had dramatically decreased. The patient was discharged with mild cerebellar ataxia on day 47. After discharge from our hospital, blood assays showed normal eosinophil count while tapering the corticosteroid dosage. Informed consent was obtained from the patient.

## 3 Discussion

The pathophysiology of EGPA-related infarctions can be attributed to multiple factors, including vasculitis, eosinophilia, and cardiac involvement [3]. This case was mainly caused by thromboembolism from endomyocarditis, but eosinophil protein-induced vascular endothelial damage and hypercoagulability might also result in multiple infarctions [4,5].

The clinical presentation differs depending on ANCA status; ANCA negativity is associated with cardiomyopathy (Table 1) ([6,7]). Among patients with EGPA-related strokes, those with cerebral hemorrhages show more vasculitic features, whereas those with cerebral infarctions frequently have cardiac involvement [2]. Cardiac involvement is pathologically divided into the necrotic stage, thrombus formation, and the fibrotic stage [8]. The first stage begins with eosinophilic infiltration of the endocardium. Thrombus formation is developed due to eosinophil protein-induced platelet activation, then thrombi along damaged endocardium are finally replaced by fibrosis.

**Table 1:** Differences in the clinical and histological features between ANCA-negative and ANCA-positive EGPA patients.

	ANCA-positive EGPA	ANCA-negative EGPA
Clinical features	Renal involvement	Cardiac involvement
	Peripheral neuropathy	Gastrointestinal manifestations
	Alveolar hemorrhage	Lung infiltrates
	Ear, nose, and throat manifestations	
Histopathological features	Vasculitis	Eosinophilic infiltration

Echocardiography is routinely performed to evaluate cardiac involvement in ischemic stroke. Delayed enhancement cardiac MRI is efficient for asymptomatic and echocardiographically unproven cases to visualize both acute inflammation and chronic fibrosis [9,10]. The notable feature of this case is that GLS obtained by tracking the endocardium detected endocardial systolic dysfunction and early left ventricular dysfunction which appeared preserved on routine echocardiography. In this case, delayed endocardial enhancement on cardiac MRI reflected irreversible fibrosis, whereas improved GLS lesions with immunosuppressive therapy indicated myocardial inflammation or edema. The evidence of embolic phenomenon and endomyocardial fibrosis suggested the progression from thrombus formation to the fibrotic stage. GLS may be a valuable tool for diagnosis and follow-up of cardiac involvement from non-invasive and sensitive perspective [11].

Corticosteroids are the basis of EGPA treatment, and the goal of treatment is to control the eosinophilia. Immunosuppressants are administered to patients with one or more poor prognostic factors defined by the Five Factor Score (FFS≧1) [12]. Cyclophosphamide is the main agent of induction therapy for severe manifestations. Hira et al. reported an autopsy case of ANCA-negative EGPA patient who developed multiple cerebral infarction due to cardiac embolism and vasculitis [3]. In this case, the patient was not administered corticosteroids since high dose steroids might worse thrombus. However, immediate steroid therapy could contribute to rapid disappearance of intraventricular thrombus associated with EGPA, which revealed the importance of reducing eosinophil levels in blood and tissue [13]. Combined immunotherapy and anticoagulation for our patient with FFS 2 were also effective in rapidly decreasing the degree of eosinophilia, controlling symptoms, preventing further complications, and improving outcome. On the other hand, there is no consensus regarding the use of anticoagulants or antiplatelets on the treatment of EGPA-related cerebral infarctions. Anticoagulant should be added for patients with hypereosinophilic syndrome who developed systemic cardioembolic events with a significant improvement in 5-year survival [14,15]. Optimal use of antithrombotic therapy should be established based on ANCA status, systemic complications, and the risk of hemorrhage.

As limitations, myocardial biopsy showed no evidence of eosinophilic infiltration or fibrosis. The utility of endomyocardial biopsy as the gold standard for diagnosis is recently reviewed in terms of limited sensitivity and technical development of cardiac imaging. In this case, negative result seemed to be due to sampling error or steroid modification [16]. Even though cardiac abnormalities were not specific, good response to immunosuppressive therapy suggested inflammatory cardiomyopathy. At least, the possibility of myocardial infarction was low because of normal findings on coronary angiography. In conclusion, ANCA-negative EGPA patients with cerebral infarctions should be carefully screened for cardiac involvement by multimodality imaging.

## Author contribution statement

All authors listed have significantly contributed to the investigation, development and writing of this article.

## Funding statement

This research did not receive any specific grant from funding agencies in the public, commercial, or not-for-profit sectors.

## Data availability statement

Data associated with this study has been deposited at https://data.mendeley.com/datasets/y6np2x9yvc/1.

## Declaration of interest’s statement

The authors declare no competing interests.
